# Morphological Asymmetries Profile and the Difference between Low- and High-Performing Road Cyclists Using 3D Scanning

**DOI:** 10.3390/biology10111199

**Published:** 2021-11-18

**Authors:** Samo Rauter, Jozef Simenko

**Affiliations:** 1 Faculty for Sport, University of Ljubljana, 1000 Ljubljana, Slovenia; samo.rauter@fsp.uni-lj.si; 2 Essex Pathways Department, University of Essex, Colchester CO4 3SQ, UK

**Keywords:** road cycling, morphological asymmetries, 3D body scanning, competition performance

## Abstract

**Simple Summary:**

There is a necessity to measure body asymmetries in road cycling as it can directly impact the performance level. The present study aimed to identify the morphological asymmetry profile of road cyclists. This study uses a novel 3D scanning method and electrical bioimpedance to investigate the impact of possible morphological asymmetries on performance in road cycling. The findings indicate that high-performance road cyclists are more symmetrical and have fewer morphological asymmetries than low-performance road cyclists.

**Abstract:**

The aims of this study are: (1) to identify morphological asymmetries in road cycling by using a novel 3D scanning method and electrical bioimpedance, (2) to investigate possible asymmetries in road cyclists of low (LPG) and high (HPG) performance group, (3) to compare the number of morphological asymmetries between HPG and LPG of cyclists, and (4) to explore correlations between asymmetry scores and competition performance. Body composition and 3D anthropometric measurements were conducted on 48 top-level male road cyclists (178.98 ± 5.39 cm; 68.37 ± 5.31 kg) divided into high (*n* = 22) and low (*n* = 26) performance groups. Competition performance (CP) is represented through racing points gathered at the end of the competition season. The latter was used to divide road cyclists into low- and high-performing groups. One-way ANOVA was used to determine differences between groups, while paired-samples T-test and Absolute Asymmetry index (AA) were calculated (*p* ≤ 0.05) for paired variables inside the groups, and the Spearman correlation coefficient was used to explore correlations between AA and CP. Results showed statistically significant differences between the left and right side of different body segments (16 paired variables) among low-performing road cyclists in five paired variables of the upper body: elbow girth (4.35, *p* = 0.000), forearm girth (6.31, *p* = 0.000), arm surface area (2.54, *p* = 0.018), and arm volume (2.71, *p* = 0.012); and six paired variables of the lower body: leg lean mass (5.85, *p* = 0.000), leg length (3.04, *p* = 0.005), knee girth (4.93, *p* = 0.000), calf girth (5.25, *p* = 0.000), leg surface area (4.03, *p* = 0.000), and leg volume (5.3, *p* = 0.000). Altogether, the high-performing group of road cyclists statistically differed only in 2 out of 16 paired variables of the upper body: elbow girth (4.93, *p* = 0.000) and in forearm girth (5.12, *p* = 0.000). Low- and high-performing groups were statistically significantly different in the asymmetry of leg lean mass *F*(1,46) = 6.25, *p* = 0.016 and asymmetry of the calf girth *F*(1,46) = 7.44, *p* = 0.009. AA of calf girth on the total sample (*n* = 48) showed a significant correlation with CP (*r* = −0.461; *p* = 0.001). In conclusion, the study’s main finding was that high-performance road cyclists are more symmetrical than the low-performance group, for which it is significant to have a higher amount of morphological asymmetries.

## 1. Introduction

Professional road cycling represents an extreme endurance sport. Elite athletes cycle approximately 30,000 to 35,000 km each year in training and competition, with some races, such as the Tour de France, lasting for 21 days and covering more than 3500 km [1]. They perform on a great variety of terrains (i.e., level vs. uphill roads) and competitive situations (i.e., individual cycling or drafting behind numerous cyclists) [2].

Various anthropometric characteristics, training characteristics, and physiological variables have been identified as significant predictors for race performance in road cycling [3]. For example, anthropometric characteristics, such as sums of particular skin folds, were shown to affect road cyclists’ split times, overall race time, and cycling speed [4,5]. It was shown that reducing body fat values between competition seasons could positively affect performance [6]. Moreover, greater body mass was connected to overall better performance: in absolute terms, larger cyclists’ frontal drag may instantly be seen as a disadvantage; however, relative to body mass, the frontal drag of smaller cyclists is considerably greater than that of large cyclists. Additionally, it was reported that the advantage does not make up for the difference in frontal drag (energy cost) to smaller cyclists with respect to relative maximum oxygen uptake (energy supply), making smaller cyclists disadvantaged in flat time trials but benefiting from it in the climbing stages [7].

In cycling, athletes specialise in different disciplines like sprint, pursuit, uphill, time trial, flat terrain, and all terrain [2,8,9,10,11]. Individual morphological characteristics [body mass, height, body surface, and frontal areas, body mass index (BMI)] partly determine a cyclist’s speciality in competition terrain [2], making anthropometric variables greatly dependent on each cyclist’s speciality [1]. Cyclists specialising in flat terrain stages tend to reduce their frontal area per body mass to improve performance during flat stages, minimising relative energy costs to aerodynamic resistance [10] and are usually taller and heavier (180 to 185 cm tall, weighing 70 to 75 kg, BMI of ~22) [2,9]. However, cyclists categorised as specialist road climbers pursue a low body mass to enhance their uphill performance, as body mass increases the resistance from gravity [10], and are usually shorter with a height of 175 to 180 cm, weighing 60 to 66 kg, with BMI of 19–20 [2,9].

Morphological research has been widely used in road cycling [11,12,13,14,15,16]. As one of the sports with repetitive movements, cycling can cause muscle force and/or flexibility asymmetries [17,18], leading to morphological asymmetries [14]. Bilateral differences are frequently found in road cycling [18,19] and can vary with the competitive situation, pedalling cadence, exercise intensity, and exercise duration [20,21,22].

There is a necessity to measure body asymmetries in road cycling as research showed that as the age of a cyclist increases, there is a tendency to increase asymmetries between the left and right sides of several body segments [14]. Morphological research also showed that cyclists have increased lower body lean mass and areal bone mineral density asymmetries than non-cyclists [23]. Asymmetrical muscle work was reported to cause different types of overloads, which can lead to injuries and deformations [24,25]. Moreover, morphological asymmetries can also negatively impact competition performance, as shown in several sports like swimming [26], track and field [27,28], and rowing [29]. Nowadays, in the literature, several different equations are being used to calculate asymmetries. However, it is difficult to ultimately justify which method should be used over another for different sports [30]. It depends on the methodology used and if we look at asymmetries as directional asymmetries, antisymmetry, fluctuating asymmetry, or sporting asymmetry [31]. From the latter, various equations can be chosen that do or do not account for the directionality of the asymmetry in paired variables.

Competition performance in cycling can be measured in different ways. For example, we could measure competition performance by the final standings after the race and categorise them as winners, podium finishers, and top 10 finishers [32]. Competition performance can also be measured by general classification by time, general classification by points, general climber’s classification, young rider classification, etc. [33]. One of the competition performance measurements in road cycling is the international ranking list (UCI ranking) or national ranking list, which has not been widely used in cycling research [11,34]. However, world ranking lists or national ranking lists have been frequently used to measure performance in a wide variety of sports like table tennis [35], judo [36,37,38,39], cross country [40], fencing [41], alpine skiing [42,43], and tennis [44].

Overall, there is a lack of research in road cycling regarding how morphological asymmetries are connected with competition performance, especially with the use of modern technology, like 3D body scanners, which have made the acquisition of data more practical, contactless, fast, and, above all, accurate [45,46].

Therefore, using the UCI ranking list as a proxy for competitive performance, we hypothesise that the high-performance group (HPG) of road cyclists will possess fewer morphological asymmetries when compared to the low-performance group (LPG). From this proposition, the study aims were (1) to identify morphological asymmetries in road cycling by using a novel 3D scanning method and electrical bioimpedance, (2) to investigate possible asymmetries in road cyclists with a specific focus on differentiating between LPG and HPG, (3) to compare the number of morphological asymmetries between HPG and LPG of cyclists, and (4) to explore correlations between asymmetry scores and competition performance.

## 2. Materials and Methods

### 2.1. Study Design

A cross-sectional research design was used to examine morphological characteristics of road cyclists with electrical bioimpedance and a 3D body scanner. First, the body composition measurements were performed in the morning (8 AM–10 AM) and followed by the 3D body scanning over two weeks in the Physiological Laboratory of the Institute of Sport in Ljubljana, Slovenia. At the end of the competitive season, the achieved racing points were recorded and used to represent the competition performance. All tests were performed and monitored by the researchers.

### 2.2. Sample

The study sample included 48 male top-level cyclists. Their mean age was 19.2 ± 2.01 years (see Table 1 for more sample characteristics). All of them were part of the Slovenian cycling federation national team and they competed at a national or international level. The main criterion was at least 3 years of training experience. The Faculty of Sport, University of Ljubljana Ethical Board (No. 10/2019) approved the study. During the study, the principles outlined in the Declaration of Helsinki were followed. Upon recruitment, a signed informed consent form was obtained from participants, and for those younger than 18 years, we obtained the consent form from their parents or guardians. At the testing time, all participants were free of acute injuries and did not report any current musculoskeletal system pain. The sample size was justified by a priori power analysis in G*power software (Version 3.1.9.7; Universität Kiel, Kiel, Germany) [47] with a type I error rate of 0.05 and 80% statistical power. Overall, the analysis indicated that 15 participants per group (total 30) are sufficient to observe significant large-sized acute effects (Cohen’s d = 0.80). Accordingly, this study involved 48 participants, 22 in the low-performance group (LPG) and 26 in the high-performance group (HPG). The division in LPG and HPG is detailed in Section 2.4 of the manuscript.

Recruited athletes were in their preparation period of training. On average, the athletes were training approximately 15–20 h per week and were on their standard dietary programs. Athletes were asked to restrain from training or any other strenuous activity one day before the testing.

### 2.3. Experimental Procedure

Anthropometric measurements were taken in the morning between 8 AM and 10 AM in an air-conditioned laboratory with the room temperature held between 21–23 °C. First, body height was measured with an anthropometer GPM (Zurich, Switzerland). Then, body composition measurements were performed using bioelectrical impedance analysis (BIA), with the InBody 720 Tetrapolar 8-Point Tactile Electrode System (Biospace Co., Ltd., Seoul, Korea). The InBody 720 apparatus utilises the technology for measuring body composition by using the method of Direct Segmental Multi-Frequency Bioelectrical Impedance Analysis. Body composition measurements were performed in the standing position, following all necessary accurate measurement guidelines [48,49]: (1) the measurements were taken in the morning (between 8 and 10 AM); (2) the participants were asked to abstain from large meals after 9 PM the evening before the test, and on the day of the measurement they neither ate nor drank before the end of the procedure; (3) participants were asked to refrain from extreme physical exertions 24 h prior to measuring, and last training should have been performed at least 12 h prior to testing; (4) the respondents did not consume alcohol 48 h before the measurement; (5) the respondents were asked to empty their bowels and bladder at least 30 min before the measurement; (6) the respondents were in the standing position for at least 5 min before the measurement to redistribute the tissue fluids; (7) the measurement was performed in the standing position by the procedure recommended by the manufacturer (hands aside placed 15 cm laterally from the body). The high test-retest, reliability, and accuracy of InBody 720 was previously assessed, with interclass correlation (ICC) reported at 0.99 [50] and correlations with the reference measure (dual-energy X-ray absorptiometry-DXA) were shown to be significant *r* = 0.95, with the reported standard error of estimate (SEE) of 1.8 [51]. With InBody 720, we measured body weight, body mass index (BMI), skeletal muscle mass, trunk lean mass, left and right arm lean mass, left and right leg lean mass, and body fat mass. Afterwards, the 3D testing took place. The pause between tests was approximately 5 min.

#### 2.3.1. The 3D Body Scan Measurements

The 3D body scanner NX-16 performed 3D anthropometric body measurement ([TC]2, Cary, NC, USA), and presents a valid [46,52] and non-invasive scanning method to produce a true-to-scale 3D body model in 8 s. Test-retest variability of the NX-16 was reported as a coefficient of variation (CV%) and ranged from 0.2–3.3% [52]. Correlations with the reference measure of manual anthropometry were shown to be significant in the range of *r* ≥ 0.95–0.99, with the average relative error in the range of 0.006–0.037 [46].

The scanner uses photogrammetry technology, which projects patterns of structured white light onto the body. Thirty-two cameras then record how the shape of the body distorts the pattern. Finally, the body shape is digitally reconstructed from raw photonic point cloud data, leading to the body’s surface reconstruction and automatic landmark recognition and electronic tape measurements. With the software, we extracted 7 single and 14 paired measurements of left and right: upper arm girth, elbow girth, forearm girth, wrist girth, arm surface area, arm volume, thigh girth, knee girth, thigh length, calf girth, shin length, leg surface area, and leg volume. Shin length was calculated as the distance between ankle height and knee joint height variables, also extracted from the 3D scan. Single measurements taken were for chest girth, waist girth, hip girth, crotch height, torso volume, torso surface area, and total body volume.

#### 2.3.2. Experimental Procedure of 3D Scanning

The subjects were measured in controlled environmental conditions by the same examiner, one with extensive experience in the physiological laboratory at the Faculty of Sport, University of Ljubljana. The scanner was located in an air-conditioned laboratory with the room temperature held between 21 and 23 °C.

Before measurements, full calibration of the NX-16 scanner was made. Full calibration was done using: (1) the reference cylinder, which was 150 cm in height and had a diameter of 28 cm, and (2) an additional set of reference balls, which included two strings of calibration balls and a single calibration ball (diameter of all balls was 15 cm). The scanner calibrated itself so that it measured a circumference on every 10 mm from the top to the bottom of the cylinder and calculated the circumferences’ standard deviation that should not have exceeded the prescribed limits of 0.9 mm [46]. Calibration with a string of balls was successful and within the acceptable range of the circumferences’ standard deviation of 0.456 mm.

Further, subjects were instructed to remove all jewellery and clothes. They entered the scanner barefooted and in form-fitting bright colour underwear. They stood in a standardised position, with their feet located on landmarks on the scanner’s floor (feet set straight, not inwards or outwards), grabbing the handles inside of the scanner with a natural standing posture (shoulders not elevated, elbows stretched, the upright position of the back, chin slightly lifted). Subjects with long hair were instructed to tie it in a bun [46].

A 3D Body Measurement System Version 7.4.1 software was used to create the initial point cloud that was then processed into a 3D body model, from which customised measurements could be extracted. A multi-scan option with three consecutive scans was used to obtain the data. Multi-scan options merged all three files of three consecutive scans and gave one merged file with all three consecutive scans. Scanning of the three consecutive scans lasted 24 s and subjects were instructed to be as still as possible [46].

### 2.4. Competition Performance

Competition performance (CP) was evaluated as racing points gathered at the end of the competitive season from national and international competitions. We used the recommended methodology for equalising national and international points as previously described by Jurov et al. [6], where international points had higher weighting and were multiplied by 2. In addition, a median approach was used to determine the threshold between LPG and HPG of road cyclists, and it was set at 100 points.

### 2.5. Statistical Analysis

Data were processed and presented using the SPSS for Windows (Version 27.0; SPSS, Inc., Chicago, IL, USA). Data were presented according to descriptive statistics (means ± SD) and 95% confidence intervals for Table 1. Furthermore, we performed the following tests: the Shapiro–Wilk test to assess the normality, a paired sample *t*-test to determine differences/asymmetries in paired body variables, and one-way ANOVA to determine differences between variables of the LPG and HPG of road cyclists. A Standardised Absolute Asymmetry (AA) score that does not account for the directionality of the asymmetry in paired variables was calculated via the formula [53,54]:

AA = (|R − L|)/(1/2(R + L)) × 100%(1)

Effect sizes (ESs) were calculated utilizing Cohen’s d. Threshold values for ES statistics were: >0.2 small, >0.5 moderate, >0.8 large, >1.3, very large [55]. The Spearman correlation coefficient was used to evaluate possible associations between CP and AA of paired variables in the LPG and HPG groups and the total sample. Statistical significance for all tests was set at *p* ≤ 0.05.

## 3. Results

Descriptive values of the sample are presented in Table 1. In addition, single body measurements of the upper body are also presented for a more complex presentation of road cyclists’ morphology (crotch height, torso volume, torso surface area, total body volume). There was a statistically significant difference between LPG and HPG of road cyclists in competition success (UCI points) as determined by one-way ANOVA *F*(1,46) = 102.2, *p* = 0.000. However, other morphological variables did not significantly differ between the groups.

The body composition and 3D anthropometric measurement of LPG and its different body segments are presented in Table 2. Altogether, the LPG road cyclists statistically differed in 10 out of 16 paired variables. Statistical significant differences between the left and right sides of different body segments among road cyclists were found in five paired variables of the upper body: elbow girth t(25) = 4.35. *p* = 0.000; forearm girth t(25) = 6.31. *p* = 0.000; arm surface area t(25) = 2.54. *p* = 0.018; and arm volume t(25) = 2.71. *p* = 0.012. Additional lower body statistical significant differences between the left and right sides of different body segments were noted: leg lean mas t(25) = 5.85. *p* = 0.000; leg length t(25) = 3.04. *p* = 0.005; knee girth t(25) = 4.93. *p* = 0.000; calf girth t(25) = 5.25. *p* = 0.000; leg surface area t(25) = 4.03. *p* = 0.000; and leg volume t(25) = 5.3. *p* = 0.000.

Body composition and 3D anthropometric measurement of the HPG and its different body segments are presented in Table 3. Altogether the HPG road cyclist statistically differed in 2 out of 16 paired variables. Statistically significant differences between the left and right sides of different body segments among road cyclists were found in two paired variables of the upper body: elbow girth t(21) = 4.93. *p* = 0.000 and forearm girth t(21) = 5.12. *p* = 0.000.

One-way ANOVA is reported in Table 4. There are no statistically significant differences between the two road cyclist groups (i.e., LPG vs. HPG) in any paired variables on the left and right body sides.

The statistically significant differences between the two road cyclist groups (i.e., LPG vs. HPG) in the asymmetry of leg lean mass *F*(1,46) = 6.25. *p* = 0.016 and asymmetry of the calf girth *F*(1,46) = 7.44. *p* = 0.009 are presented in Table 5.

The Spearman correlation coefficient showed a significant association of competition success with the asymmetry of calf girth (*r* = −0.461; *p* = 0.001) for the whole sample (*n* = 48). LPG and HPG AA scores of paired variables did not show any significant correlations with competition success.

## 4. Discussion

The present study aimed to use a novel 3D body scanning method to identify the morphological asymmetry profile of road cyclists, compare asymmetries between LPG and HPG, and investigate correlations between morphological asymmetries on competition performance in road cyclists. The main findings were: (1) 3D body scanning method is a fast and useful method to detect asymmetries; (2) HPG road cyclists are more symmetrical than the LPG; (3) the latter also exhibited a higher number of morphological asymmetries (asymmetries LPG 10/16 vs. HPG 2/16 variables); and (4) lower asymmetry of calf girth correlated with greater competition success in road cyclists.

The literature showed that cardiorespiratory testing was the most frequent procedure to assess performance in cyclists and only a few studies identified a correlation between the morphological asymmetry profile and performance. Studies explained that there is a tendency among humans to preferentially use one side of the body in a voluntary act [20]. This tendency characterises lateral preference. Lateralisation has been suggested to be only 10–20% dependent on genetics. Other influences, such as task complexity, gender, and developmental characteristics, play an important role in body side choice. Among the few studies considering the bilateral pedalling assessments, the data consistently show that cyclists present frequent asymmetry [19,56]. The amount of asymmetry can vary within subjects and the limb producing asymmetry. Moreover, the pedalling asymmetry appears to be related to limb preference and is significantly reduced with an increase of pedalling workload. It was also shown that even with a symmetrical pedal force production, the existing bilateral difference in the pedalling kinematics leads to the asymmetry in joint torques and muscle loads [57]. These pedalling asymmetries that reflect the asymmetry of joint torques and muscle loads could explain the development of morphological asymmetries shown in our study.

Comparison of the selected anthropometric characteristics (Table 1) between LPG and HPG of cyclists showed no statistical differences. Similar trends in body height and body weight were shown compared to previous studies with road cyclists [11,15]. For example, track cyclists were found to have higher body weight with more skeletal muscle mass, which might be explained by the performance characteristics of the cycling discipline [58]. We used BIA and a 3D body scanner (NX-16) to estimate body composition measurement, representing a relatively new anthropometric assessment method. To calculate asymmetries in our study, we measured 16 paired variables (lean mass of arm and leg; girth of the upper arm, elbow, forearm, wrist, thigh, knee, and calf; length of thigh and shin; leg and arm volume; leg and arm surface area). Our study demonstrates that the 3D body scanning method combined with BIA presents a valuable tool in road cycling to quickly assess body asymmetries and other morphological variables. These could help coaches identify potential morphological asymmetries and modify strength and conditioning training to lower asymmetries and increase performance.

The body asymmetries between the left and right sides of different body segments among LPG and HPG are presented in Table 2 and Table 3. Among HPG, we found statistically significant differences between left-right sides only in two upper body variables (forearm and elbow girth). However, we found a great number of statistically significant differences between the left and right sides of different body segments when examining LPG (asymmetries in 10 out of 16 paired variables). Results showed statistical differences in four paired variables of the upper body (elbow and forearm girth, arm surface area, and arm volume) and six of the lower body variables (leg lean mass, leg length, knee and calf girth, leg volume, and leg surface area). These results demonstrate that our proposition that HPG is more symmetrical than LPG is well-assumed. In addition, there were statistical differences among both groups of road cyclists between the left and right calf girth and leg lean mass index of asymmetry (Table 5). The absolute asymmetry index (AA) in the aforementioned variables was lower in the HPG, meaning they developed more symmetrically. Noted morphological asymmetries are in line with previous research [14]. However, our study is the first to report body volume and body surface area extracted from a 3D body scanner. These variables could be used as reference values for further studies, especially when connected to time trials and competition performance.

Lower body asymmetries (leg lean mass and calf girth asymmetry) can lead as a cause of possible reduced cycling power and poorer performance in cycling competitions. This is supported by our results of a negative correlation of AA of calf girth with competition success (*r* = −0.461; *p* = 0.001), meaning smaller calf girth asymmetries are correlated with better competition performance of road cyclists.

The limitations of our study were that we made a comparison of a small amount of pre-selected morphological variables. In addition, a low number of previous studies have investigated morphological asymmetries with 3D body scanners, which limited the discussion of the findings. For future research, it might be better to study different cycling disciplines according to their performance level and follow their progress from the junior to the elite level of cycling. Additionally, the question of whether these asymmetries lead to a greater occurrence of injuries or a bigger dropout, or if they can be related to better cycling performance, still needs to be further researched.

## 5. Conclusions

The study’s main findings indicated that high-performance road cyclists have fewer morphological asymmetries than low-performance road cyclists. Furthermore, due to the results in the study and some statistical differences obtained between both groups, it could be intuited that the morphological asymmetries profile could be predictors of performance in road cycling competitions. Therefore, these asymmetries could nowadays be assessed and identified in a fast and contactless manner with the usage of 3D scanners. Furthermore, these measurements could give coaches quick feedback that could be used in strength and conditioning training to lower the identified asymmetries and increase competition performance.

## Figures and Tables

**Table 1:** One-way ANOVA with the descriptive presentation of the sample single morphological measurements with means (±SD) and 95% confidence interval (CI) values.

Group	LPG			HPG				
Variable	Mean	SD	95% CI	Mean	SD	95% CI	F	Sig.
Height (cm)	178.98	5.39	177–181	178.78	5.18	177–180	0.017	0.897
Weight (kg)	68.37	5.31	66–71	67.02	7.43	64–70	0.536	0.468
BMI (kg/m^2^)	21.33	1.20	21–22	20.94	1.81	20–22	0.821	0.370
SMM (kg)	35.26	3.29	34–37	34.40	4.34	33–36	0.602	0.442
SMM (%)	51.52	1.40	51–52	51.27	1.71	51–52	0.292	0.591
Body Fat (%)	9.59	1.98	8.8–10.4	9.57	2.80	8.3–10.8	0.000	0.983
Chest Girth (cm)	97.17	3.46	96–98	96.07	4.39	96–98	0.938	0.338
Waist Girth (cm)	79.17	4.22	77–81	78.28	4.39	76–80	0.508	0.480
Hip Girth (cm)	93.53	3.19	92–95	93.10	4.04	91–95	0.174	0.679
Crotch Height (cm)	84.69	4.04	83–86	85.35	4.50	83–87	0.284	0.596
Trunk Lean Mass (kg)	27.14	2.29	26–28	26.42	3.24	25–28	0.805	0.374
Torso Volume (L)	35.80	4.20	34.1–37.4	34.91	4.88	32.7–37.1	0.458	0.502
Torso Surface Area (m^2^)	0.553	0.057	0.529–0.576	0.549	0.058	0.523–0.575	0.063	0.803
Total Body Volume (L)	61.41	4.87	59.4–63.4	60.31	6.93	57.2–63.4	0.412	0.524
Competition success (UCI points) *	35.08	33.72	22–49	254.41	104.57	208–301	102.2	0.000 *

Legend: BMI—Body mass index; SMM—skeletal muscle mass; CS—Competition success; UCI—Union Cycliste Internationale; LPG—low performance group; HPG—high performance group; SD—standard deviation; L—litres; cm—centimetres; kg—kilograms; %—percentage; m^2^—meters squared; * *p* ≤ 0.05.

**Table 2:** Mean (±SD) values for left and right morphological variables with a paired *t*-test between paired variables of the LPG of road cyclists.

	Pair	Variable	Body Side							Effect Size
			Left		Right					
			Mean	SD	Mean	SD	*df*	t	*p*	
UPPER BODY	1	Arm Lean Mass (kg)	3.45	0.39	3.48	0.38	25	1.87	0.073	0.367
	2	Upper arm Girth (cm)	29.67	1.71	29.90	1.85	25	1.3	0.204	0.256
	3	Elbow Girth (cm)	25.45	1.04	26.00	0.92	25	4.35	0.000 *	0.854
	4	Forearm Girth (cm)	26.14	0.99	26.89	0.96	25	6.31	0.000	1.237
	5	Wrist Girth (cm)	16.74	0.92	16.62	0.68	25	1.06	0.297	0.209
	6	Arm Surface Area (m^2^)	0.132	0.008	0.134	0.008	25	2.54	0.018 *	0.498
	7	Arm Volume (L)	3.65	0.29	3.13	0.31	25	2.71	0.012 *	0.531
LOWER BODY	8	Leg Lean Mass (kg)	9.63	0.97	9.74	0.98	25	5.85	0.000 *	1.147
	9	Leg Length (cm)	104.34	4.88	104.46	4.87	25	3.04	0.005 *	0.597
	10	Thigh Length (cm)	34.37	4.22	34.40	4.18	25	0.54	0.594	0.106
	11	Thigh Girth (cm)	61.68	5.69	62.18	5.82	25	1.02	0.319	0.199
	12	Knee Girth (cm)	39.80	2.35	40.37	2.66	25	4.93	0.000 *	0.968
	13	Shin Length (cm)	42.83	3.88	42.85	3.89	25	0.83	0.416	0.162
	14	Calf Girth (cm)	37.67	1.50	38.27	1.48	25	5.25	0.000 *	1.030
	15	Leg Surface Area (m^2^)	0.311	0.024	0.314	0.025	25	4.03	0.000 *	0.790
	16	Leg Volume (L)	9.63	0.92	9.85	1.02	25	5.3	0.000 *	1.040

Legend: SD—standard deviation; L—litres; cm—centimetres; kg—kilograms; m^2^—meters squared; * *p* ≤ 0.05.

**Table 3:** Mean (±SD) values for left and right morphological variables with a paired *t*-test between paired variables of the HPG of road cyclists.

	Pair	Variable	Body Side							Effect Size
			Left		Right					
			Mean	SD	Mean	SD	*df*	t	*p*	
UPPER BODY	1	Arm Lean Mass (kg)	3.33	0.55	3.36	0.57	21	1.269	0.218	0.270
	2	Upper arm Girth (cm)	29.76	2.08	29.65	2.25	21	0.414	0.683	0.088
	3	Elbow Girth (cm)	25.40	1.81	26.15	1.71	21	4.929	0.000 *	1.051
	4	Forearm Girth (cm)	25.94	17.16	26.58	1.84	21	5.122	0.000 *	1.092
	5	Wrist Girth (cm)	17.01	0.98	17.00	0.76	21	0.036	0.972	0.008
	6	Arm Surface Area (m^2^)	0.134	0.012	0.135	0.013	21	1.110	0.280	0.237
	7	Arm Volume (L)	3.10	0.47	3.11	0.49	21	0.310	0.760	0.066
LOWER BODY	8	Leg Lean Mass (kg)	9.65	1.08	9.68	1.10	21	1.087	0.290	0.232
	9	Leg Length (cm)	104.96	4.66	105.02	4.53	21	1.105	0.281	0.236
	10	Thigh Length (cm)	34.43	3.73	34.46	3.69	21	0.560	0.582	0.119
	11	Thigh Girth (cm)	60.51	6.49	59.78	6.14	21	1.588	0.127	0.339
	12	Knee Girth (cm)	40.13	2.99	40.41	3.18	21	2.033	0.055	0.433
	13	Shin Length (cm)	43.35	3.70	43.40	3.75	21	1.482	0.153	0.316
	14	Calf Girth (cm)	37.90	2.02	38.14	2.25	21	1.804	0.086	0.385
	15	Leg Surface Area (m^2^)	0.310	0.029	0.311	0.031	21	0.871	0.394	0.186
	16	Leg Volume (L)	9.59	1.33	9.68	1.33	21	1.785	0.089	0.381

Legend: SD—standard deviation; L—litres; cm—centimetres; kg—kilograms; m^2^—meters squared; * *p* ≤ 0.05.

**Table 4:** One-way ANOVA with the descriptive presentation of the morphological variables between the LPG and HPG for the left and right body sides.

Variable	Group	Body Side							
		Left				Right			
		Mean	SD	F	Sig.	Mean	SD	F	Sig.
Arm Lean Mass (kg)	LPG	3.45	0.39	0.671	0.417	3.48	0.38	0.755	0.390
	HPG	3.33	0.55			3.36	0.57		
Upper Arm Girth (cm)	LPG	29.66	1.71	0.038	0.847	29.90	1.85	0.173	0.679
	HPG	29.76	2.08			29.65	2.25		
Elbow Girth (cm)	LPG	25.45	1.04	0.014	0.905	26.00	0.92	0.158	0.693
	HPG	25.40	1.81			26.15	1.71		
Forearm Girth (cm)	LPG	26.14	0.99	0.259	0.613	26.89	0.96	0.581	0.450
	HPG	25.93	1.72			26.58	1.84		
Wrist Girth (cm)	LPG	16.74	0.92	0.912	0.345	16.62	0.68	3.242	0.078
	HPG	17.01	0.98			17.00	0.76		
Arm Surface Area (m^2^)	LPG	0.132	0.008	0.469	0.497	0.134	0.008	0.175	0.678
	HPG	0.134	0.012			0.135	0.012		
Arm Volume (L)	LPG	3.07	0.29	0.075	0.786	3.13	0.31	0.050	0.824
	HPG	3.10	0.47			3.11	0.49		
Leg Lean Mass (kg)	LPG	9.63	0.97	0.006	0.937	9.74	0.98	0.046	0.830
	HPG	9.65	1.08			9.68	1.10		
Leg Length (cm)	LPG	104.34	4.88	0.204	0.653	104.62	4.87	0.166	0.686
	HPG	104.96	4.56			105.18	4.53		
Thigh Length (cm)	LPG	34.37	4.22	0.003	0.958	34.40	4.18	0.003	0.958
	HPG	34.43	3.73			34.46	3.69		
Thigh Girth (cm)	LPG	61.68	5.69	0.447	0.507	62.18	5.82	1.925	0.172
	HPG	60.51	6.49			59.78	6.14		
Knee Girth (cm)	LPG	39.80	2.35	0.180	0.673	40.37	2.66	0.002	0.962
	HPG	40.13	2.99			40.41	3.18		
Shin length (cm)	LPG	42.83	3.88	0.226	0.637	42.85	3.89	0.243	0.625
	HPG	43.35	3.70			43.40	3.75		
Calf Girth (cm)	LPG	37.67	1.50	0.191	0.664	38.27	1.48	0.056	0.814
	HPG	37.90	2.02			38.14	2.25		
Leg Surface Area (m^2^)	LPG	0.312	0.024	0.059	0.809	0.314	0.025	0.177	0.676
	HPG	0.310	0.029			0.311	0.031		
Leg Volume (L)	LPG	9.63	0.92	0.016	0.901	9.85	1.02	0.272	0.605
	HPG	9.59	1.33			9.68	1.33		

Legend: SD—standard deviation; L—litres; cm—centimetres; kg—kilograms; m^2^—meters squared.

**Table 5:** One-way ANOVA with the descriptive presentation of the Absolute Asymmetry Index (AA) of paired morphological measurements of LPG and HPG with means (±SD), 95% confidence interval (CI) values and effect sizes.

Variable	G	Mean	SD	95% CI		F	Sig.	EF
				Lower	Upper			
AA Arm Lean mass	LPG	2.26	1.72	1.56	2.95	0.086	0.771	0.002
	HPG	2.42	2.05	1.51	3.32			
AA Upper Arm Girth	LPG	2.51	1.85	1.76	3.26	2.514	0.120	0.052
	HPG	3.45	2.27	2.45	4.46			
AA Elbow Girth	LPG	2.60	2.09	1.76	3.45	1.729	0.195	0.036
	HPG	3.43	2.26	2.43	4.43			
AA Forearm Girth	LPG	3.03	2.07	2.19	3.86	0.173	0.680	0.004
	HPG	2.79	1.77	2.01	3.58			
AA Wrist Girth	LPG	2.56	2.07	1.72	3.40	0.034	0.855	0.001
	HPG	2.67	2.23	1.68	3.66			
AA Arm Surface Area	LPG	2.00	1.54	1.37	2.62	0.157	0.693	0.003
	HPG	2.17	1.45	1.53	2.81			
AA Arm Volume	LPG	3.10	3.21	1.80	4.39	0.212	0.647	0.005
	HPG	3.50	2.71	2.29	4.70			
AA Leg Lean Mass	LPG	1.29	0.79	0.97	1.61	6.246	0.016 *	0.120
	HPG	0.76	0.65	0.48	1.05			
AA Leg Length	LPG	0.19	0.14	0.13	0.25	0.095	0.759	0.002
	HPG	0.17	0.20	0.09	0.26			
AA Thigh Length	LPG	0.65	0.61	0.41	0.90	0.409	0.526	0.009
	HPG	0.76	0.52	0.53	0.99			
AA Thigh Girth	LPG	2.86	2.66	1.79	3.94	0.067	0.797	0.001
	HPG	2.66	2.60	1.51	3.82			
AA Knee Girth	LPG	1.67	1.08	1.24	2.11	1.508	0.226	0.032
	HPG	1.30	1.03	0.84	1.75			
AA Shin Length	LPG	0.12	0.31	0.00	0.24	0.525	0.472	0.011
	HPG	0.18	0.29	0.06	0.31			
AA Calf Girth	LPG	1.98	0.91	1.61	2.35	7.440	0.009 *	0.139
	HPG	1.10	1.30	0.52	1.68			
AA Leg Surface Area	LPG	1.04	0.77	0.73	1.35	2.278	0.138	0.047
	HPG	1.46	1.16	0.95	1.97			
AA Leg Volume	LPG	2.34	2.038	1.51	3.16	0.938	0.338	0.20
	HPG	1.78	1.938	0.92	2.64			

Legend: AA—absolute asymmetry index; G—group; SD—standard deviation; EF—effect size; * *p* ≤ 0.05

## Data Availability

The data that support the findings of this study are available from the corresponding author upon reasonable request.
